# In defence of *Bacillus thuringiensis*, the safest and most successful microbial insecticide available to humanity—a response to EFSA

**DOI:** 10.1093/femsec/fix084

**Published:** 2017-06-22

**Authors:** Ben Raymond, Brian A. Federici

**Affiliations:** 1 Environmental Sustainability Institute and Department of Biosciences, University of Exeter, Penryn Campus, Penryn TR10 9FE, UK; 2 Department of Entomology and Institute for Integrative Genome Biology, University of California, Riverside, Riverside, CA 92521, USA

**Keywords:** *Bacillus cereus* group, biocontrol, biopesticide, food safety, phylogeny

## Abstract

The *Bacillus cereus* group contains vertebrate pathogens such as *B. anthracis* and *B. cereus* and the invertebrate pathogen *B. thuringiensis (Bt)*. Microbial biopesticides based on *Bt* are widely recognised as being among the safest and least environmentally damaging insecticidal products available. Nevertheless, a recent food-poisoning incident prompted a European Food Safety Authority review which argued that *Bt* poses a health risk equivalent to *B. cereus*, a causative agent of diarrhoea. However, a critical examination of available data, and this latest incident, provides no solid evidence that *Bt* causes diarrhoea. Although relatively high levels of *B. cereus*-like spores can occur in foods, genotyping demonstrates that these are predominantly naturally occurring strains rather than biopesticides. Moreover, MLST genotyping of >2000 isolates show that biopesticide genotypes have never been isolated from any clinical infection. MLST data demonstrate that *B. cereus* group is heterogeneous and formed of distinct clades with substantial differences in biology, ecology and host association. The group posing the greatest risk (the *anthracis* clade) is distantly related to the clade containing all biopesticides. These recent data support the long-held view that *Bt* and especially the strains used in Bt biopesticides are very safe for humans.

## Teaser

This article critically examines the available evidence on whether *Bacillus thuringiesis*, a species commericially important in biocontrol, is capable of infecting vertebrates.

## INTRODUCTION

Let us begin with a thought experiment. What would we, as scientists and regulators, like to know in order to be able confidently recommend that a microbial control agent is safe for application to growing crops? We would need to be confident that the key active component of our biopesticide has no opportunity to interact with receptors on human cells. We would like to know that these microbes are not able to infect vertebrates orally, by inhalation or via injection. We would prefer that our microbe of choice did not associate with humans, even commensally, and it would be better if its biology and ecological niche were well described. If we were particularly cautious, we might like to hold off giving a firm scientific opinion until such a product had been used in the field for a number of years, perhaps for many decades.

**Figure 1.**
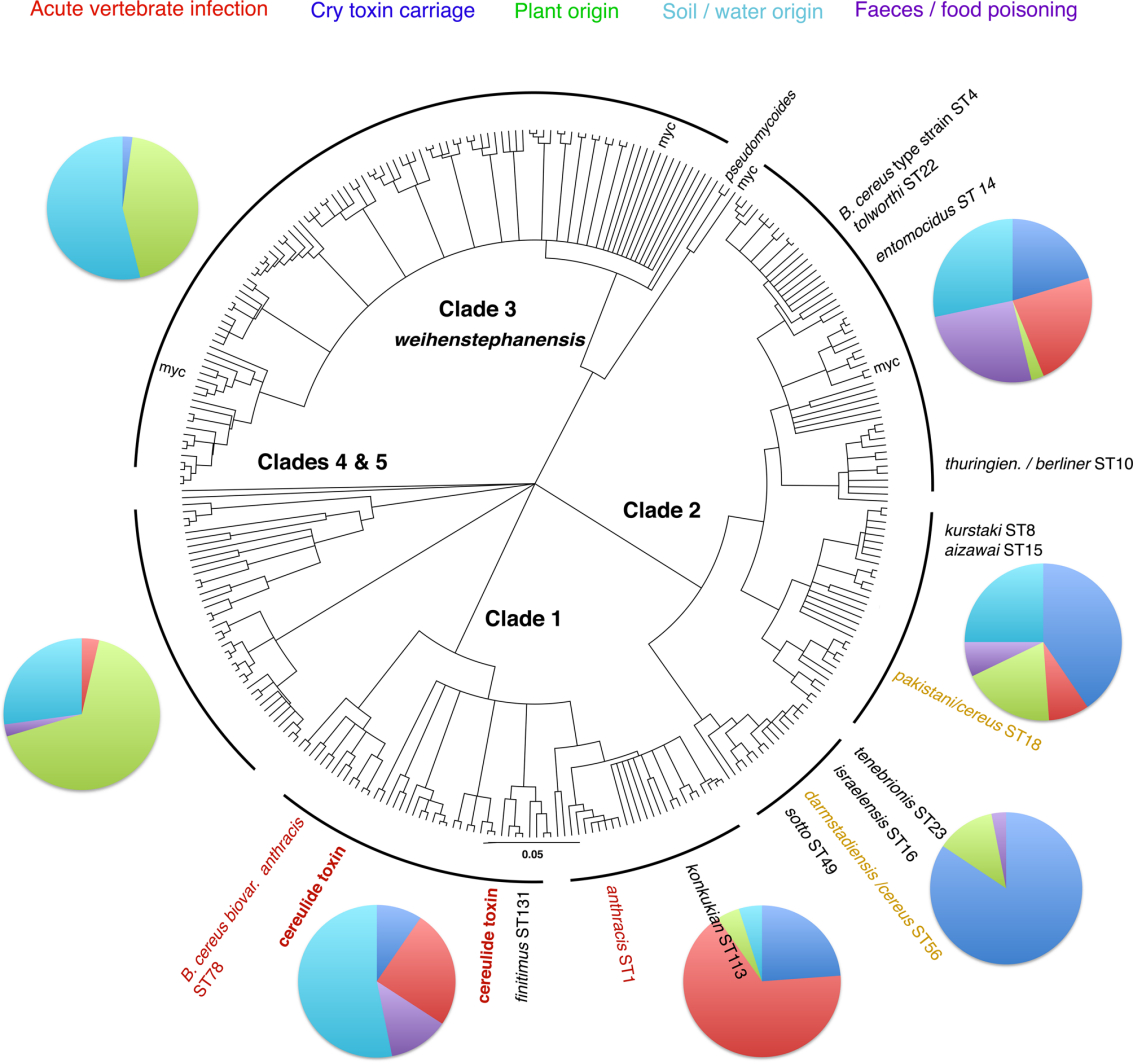
Variation in ecology, host and habitat association across distinct clades in the *B. cereus* group. The phylogeny was based on the Priest *et al.* (2004) MLST scheme and constructed using CLONALFRAME and redrawn from the analysis in the study by Raymond *et al.* (2010b). The pie charts describe the proportion of isolates with particular host-associated (Cry toxins, pXO1/XO2 plasmids) traits or their habitat and host of origin; the colour-coded text at the top of the figure explains the affiliations; the arcs in bold black describe the extent of the clades or subclades. Pie charts summarise data for the two major subgroups of clade 1 and three major groups of clade 2. Key vertebrate and invertebrate-associated STs are indicated around the tree; red indicates vertebrate association. STs labelled in gold with a serovar and the epithet *cereus* are genotypes that have been described as both *B. cereus* and *B. thuringiensis;* ST14 is also the *B. cereus* type strain*.* The abbreviation ‘myc’ refers to genotypes that have been described as *B. mycoides.* Clade 1 corresponds to cluster III in the Guinebretière *et al.* (2010) scheme, clade 2 to cluster IV and clade 3 to cluster II and *pseudomycoides* indicates the position of cluster I.

For the world's best-selling microbial pesticide, *Bacillus thuringiensis* (*Bt*), we have all this information (Siegel 2001; Federici and Siegel 2007). Not only is *Bt* safe for vertebrates but a number of reviews, including an IOBC/WPRS working group, have concluded that *Bt* is also one of safest products available in terms of impacts on non-target insects (Hassan 1992; Glare and O’Callaghan 2000). *Bt* is therefore an important environmentally friendly part of the modern pest management tool kit. The only other group of pesticides that may be safer are baculovirus products, which typically have a very narrow host range such as an insect genus or species (Huber 1988), and constitute a minor market because they must be produced in caterpillars. Despite decades of accumulated biological, ecological and safety data, the use of *Bt* is now under threat in Europe. Significantly, this change of heart on the part of the European regulators (European Food and Safety Authority, EFSA) is not based on new scientific evidence, but rather on an isolated and highly publicised incident of food poisoning in Germany, in which *Bt* was *not* identified as the etiological agent with any degree of reliability (EFSA 2016). This isolated case led to a new working group, which reached the contentious, and in our view erroneous conclusion that *Bt* is biologically and ecologically equivalent to *Bacillus cereus (Bc)*, a known cause of human food poisoning (Granum and Lund 1997; Stenfors Arnesen, Fagerlund and Granum 2008), since both are close relatives in the *Bc* group. Currently, *Bt* and *Bc* are typically distinguished using a single phenotypic character, the production of inclusion bodies composed of crystal (Cry) proteins encoded by genes on large plasmids (Gonzalez, Dulmage and Carlton 1981). Many (but not all) *Bt* strains are chromosomally similar to strains of *Bc* (Fig. 1) (Raymond and Bonsall 2013)*.* Notably, the presence of absence of *cry* genes is not a very reliable indicator of how strains have been classified (Liu *et al.*^2015^), either because of the presence of pseudogenes or because the phenotypic definition has not been carefully applied. Nevertheless, the ecological distinctiveness of *Bt* as a group of specialised invertebrate pathogens has been widely accepted by most experts and regulatory agencies for decades (Raymond *et al.*2010a; Ruan *et al.*^2015^) and is formally recognised through the different hazard levels assigned to each species: biohazard level 1 (for *Bt*) and biohazard level 2 (for *Bc* as an opportunistic pathogen). Thus, arguments over the taxonomic status of *Bt* and its ecological and biological identity have been a major cause of this latest controversy.

In addition, the recent EFSA opinion encompassed a broad, but uncritical review of the *Bt* and *Bc* literature and came to conclusions about the ability of *Bt* to infect humans that differ markedly from previous analyses (Glare and O’Callaghan 2000; Siegel 2001; Federici and Siegel 2007). Importantly, they make the poorly substantiated claim that we are largely ignorant of the ability of *Bt* to infect vertebrates and thus should treat it as of equivalent risk to humans as *Bc* based on the precautionary principle. Here, we challenge the conclusion that we are ignorant of *Bt’*s biology*;* point out new evidence supporting its safe track record that has appeared since the last major reviews of this topic; show how the ESFA Opinion article distorts the data on *Bt* strains in foods and biopesticides implying it may be a significant cause of food poisoning; and dispute the idea that the *Bc* group is biologically and ecologically homogeneous.

### How the trouble started—from molehill to mountain

The details of the case of food poisoning that prompted the recent EFSA enquiry are worth repeating. In July 2012, a German family of five ate a meal of cheese noodles; three members of the family also ate a salad and these three members became ill with diarrhoea (EFSA 2016). Food samples were analysed for presumptive causative agents: the salad contained 3 × 10^4^ CFU g^−1^ of *Bt*; the cheese noodles contained 6.0 × 10^3^ CFU g^−1^ of *Bc.* The *Bt* was identified as being indistinguishable from the *Bt aizawai* strain that is the active ingredient of the biopesticide XenTari applied to the salad crops in question. Repeat sampling of the salad from the supermarket where the original product was purchased found *Bt* concentrations of 4 × 10^4^ and 1.5 × 10^5^ CFU g^−1^*.*

This level of evidence cannot reliably implicate *Bt* as the cause of infection as there are two possible etiological agents involved. However, we can use the scientific literature to assess the balance of probabilities in favour of one agent or another. Is the fact that three of five individuals became ill after consuming cheese noodles contaminated with *Bc* consistent with *Bc* being the causative agent? Estimates of the infective dose of *Bc* required to establish an infection vary widely but commonly cover the 10^5−^10^7^ CFU g^−1^ range (Stenfors Arnesen, Fagerlund and Granum 2008). Nevertheless, concentrations as low 10^2^–10^3^ in food have been found associated with disease (Stenfors Arnesen, Fagerlund and Granum 2008). Moreover, basic epidemiological principles assert that there is no one ‘infective dose’ for pathogens, but a dose-response curve in which increasing doses lead to a higher probability of infection, which is well described for *Bt* (Cornforth *et al.*^2015^). Even if 10^3^–10^4^ CFU g^−1^ constitutes a low dose of *Bc*, an infection rate of 60% is entirely consistent with what we know of this organism and of epidemiology in general. The fact that the three individuals who became ill also happened to eat the salad could therefore be a coincidence. Using simple probability theory, we can calculate precisely how much of a coincidence this was. There are 5!/(3!(5–3)! ways, i.e. only 10 ways, of choosing three individuals from a family of five. In only one out of these 10 combinations would all three infected individuals be the same individuals who ate the salad: giving us a probability of 0.1. In the scale of unlikely events, a probability of 10% is a rather ordinary coincidence, one that would not meet the bar of statistical significance, and which is particularly modest given that tens of thousands of families across Europe eat tomatoes and other vegetables that have been sprayed with *Bt* products (Rosenquist *et al.*^2005^; Frederiksen *et al.*^2006^)*.* Direct evidence to implicate *Bt* rather than *Bc* as a cause of diarrhoea, for example, evidence of *Bt* proliferation from stool samples, was not provided.

### Safety testing of Bt in vertebrates

On the other hand, how likely is it that the alternative hypothesis is correct, i.e. that *Bt aizawai* was actually the cause of infection in the case above? Leaving the debate on whether *any* strains of *Bt* can cause vertebrate infections until later, let us first consider whether *‘*biopesticidal’ strains of *Bt* can cause infections in humans. Here, we will largely summarise the main points from previous reviews of the biosafety of *Bt* (Siegel 2001; Federici and Siegel 2007). For instance, between 1961 and 1995, the United States Environmental Protection Agency licensed 177 products that used *Bt* spores and Cry crystals as active ingredients; all were tested for infectivity in mammals (McClintock, Schaffer and Sjoblad 1995; Siegel 2001). While licensed products can cause mortality in vertebrates at very high doses, there is a threshold dose above which pathogens are considered safe. In the USA, this is a dose of 10^6^ spores into a mouse. However, in general, doses of *Bt* required to kill small mammals by injection/gavage are typically greater than 10^8^ spores, which for humans would be equivalent to a dose in the region 10^11^ spores (Siegel 2001). To put this in perspective, that would be the dose found on ∼10 000 kg of the salad in the above German food-poisoning incident.

Notably, it is hard to find evidence of oral doses of *Bt* biopesticides that are high enough to cause any infection or other symptoms in vertebrates. Rats fed 10^9^ spores per day for 730 days successfully suffered no ill effects (Siegel 2001); doses of 10^12^ have no effects on sheep or rats (Siegel 2001). The rat study in particular assessed six different strains over 3 weeks. A concentration of 10^10^ CFU ml^−1^ does not affect mice (Berlitz *et al.*^2012^) and over 5 days human volunteers can consume 1 g per day (∼ 10^11^ spores) of a formulated product (Thuricide) based on *Bt* without ill effects (Siegel 2001). Epidemiological studies confirm the results of acute toxicity tests. The city-wide application of *Bt* to Auckland in New Zealand and to Victoria in British Columbia did not result in detectable impacts on health problems in comparison to unsprayed areas of those cities, although elevated levels spores of *Bt kurstaki* could be recovered from the nasal swabs of inhabitants, confirming that there had been exposure (Federici and Siegel 2007).

### The occurrence of *Bt and Bc* in food and the environment

Given the above experimental studies on the safety of *Bt* to vertebrates, it is relevant to note the rates and composition of *Bt* products used commonly to control caterpillar pests in organic agriculture and integrated pest management programmes. Unlike most synthetic chemical insecticides, *Bt* biopesticides can be applied as late as the day before harvest because of their record of safety. Products such as Biobit, Dipel, Foray and Thuricide, as well as many others used in different countries around the world are based on the HD-1 isolate of *Bt kurstaki*. In addition to viable spores, these products contain four insecticidal proteins: Cry1Aa, Cry1Ab, Cry1Ac and Cry2Aa (Crickmore *et al.*^1998^; Schnepf *et al.*^1998^). Similarly, most commercial *Bt aizawai* products are based on the HD-133 or a similar isolate, which contains viable spores and four Cry proteins: Cry1Aa, Cry1Ac, Cry1C and Cry1D (Kuo and Chak 1996). The concentrations of viable spores in products based on the above *Bt kurstaki* and *aizawai* strains are typically in the region of 10^6^ mg^−1^. Biopesticide label recommendations state that these can be sprayed at coverage rates from 0.01 to 0.1 mg per cm^2^, or in other words, about 10 000 to 100 000 spores per cm^2^ of crop surface area. In addition to the active ingredients, commercial products contain dried spent fermentation media, and protective efficacy enhancers, commonly referred to as UV protectants, and spreaders and stickers to enhance adherence of the spores and Cry crystals to crops so they are not washed off by rain or overhead irrigation. Many vegetables including tomatoes, celery and cucumbers are eaten raw, and if sprayed with *Bt* products, these adherence enhancers make it difficult to wash the spores and Cry crystals off the crop. Thus, it is not surprising that CFUs in the range of 10^3^–10^6^ gm^−1^ are found on fresh vegetables in supermarkets. Even given the possible effects of vegetable washing, if consuming up to 10^6^ gm^−1^*Bt* spores caused diarrhoea we would expect at least people who consume organic crops to routinely report this illness; however, there are no data supporting this.

The EFSA Opinion paper attempted to raise questions about the safety of *Bt* and *Bt* biopesticides by focusing on data in two peer-reviewed studies that deal with the occurrence of *Bt* in food (Rosenquist *et al.*^2005^; Frederiksen *et al.*^2006^). Two other studies dealing with a limited number of food-poisoning events in which *Bt* was implicated as the causative agent are discussed below (Jackson *et al.*^1995^; McIntyre *et al.*^2008^). The data reviewed are accurate. However, the interpretations are misleading, if not wrong, with respect to the source of the *Bt* strains identified—naturally occurring or from Bt biopesticides—and whether the latter actually caused disease. In this regard, the data published by Rosenquist *et al.* (2005) and Frederiksen *et al.* (2006) on ready-to-eat foods in Danish markets are relevant. In the Rosenquist *et al.* (2005) study, 0.5% of food samples had counts of *Bt/Bc* higher than 10^4^ CFU/g, a level considered unacceptable for human consumption under Danish guidelines. These high counts were found in fresh tomatoes, cucumbers and heat-treated ready-to-eat starchy foods, especially desserts containing rice, nuts and milk. Of 40 strains tested for parasporal Cry crystals and *cry* genes, 31 were positive, allowing these to be identified as *Bt*, and all contained genes for protein enterotoxins that could cause diarrhoea. Based on these results, the authors concluded ‘These observations indicate that *B. thuringiensis* could actually be responsible for many of the food borne outbreaks here previously attributed to *B. cereus sensu stricto*’. This conclusion is misleading primarily because there is no evidence for food poisonings resulting from high counts of *Bt* in food.

It would also be erroneous for a regulator to infer from these studies that limiting spray residues on crops would substantially reduce the exposure to *Bt* in food. For example, only 5 of the 40 strains (12.5%) tested had profiles characteristic of *Bt* biopesticides. Another flaw in the study is that the PCR tests only screened for two cry genes (*cry1Aa* and *cry1Ab*) that occur widely in many natural *Bt* isolates (Crickmore *et al.*^1998^) so these tests are not sensitive enough to reliably identify any strain as having a biopesticidal origin. In the follow-up study, Frederiksen *et al.* (2006) used plasmid and *cry* gene profiles to determine if *Bc* group strains had genotypes identical to those of *Bt* biopesticides. Fredriksen *et al.* (2006) found that 18% of the 128 isolates had plasmid profiles characteristic of *Bt* strains used in commercial products. When these genotypes were present in high concentrations (CFU > 10 000 g^−1^), these strains originated from cucumber or cherry tomatoes. These are not starchy foods in which spores are likely to germinate or in which vegetative cells are prone to multiply, and thus are highly unlikely to result in food poisoning. More importantly with respect to use of *Bt* biopesticides, these data show that between 80% and 90% of isolates were from natural isolates of *Bt* rather than biopesticide strains (Rosenquist *et al.*^2005^; Frederiksen *et al.*^2006^). What is not mentioned in the EFSA Opinion paper is the common occurrence of a wide array of *Bt* strains in all kinds of stored grains and nuts (Burges and Hurst 1977; Meadows *et al.*^1992^; Itova-Aoyolo *et al.*^1995^) most of which do not have the specific gene profiles of *Bt* biopesticides. Thus, grains and nuts and dusts from storage granaries are the probable source of the naturally occurring *Bt* strains commonly found in ready-to-eat foods studied from Danish markets.

### Bt and human infections: case studies and new epidemiological data

What then if we cast our net more broadly, is there evidence that any strain of *Bt* can cause gastrointestinal or tissue infections in vertebrates. The number of infections in humans where *Bt* strains are a clear causative agent is extremely few, if any. *Bt* has been recovered from infected burn wounds (Damgaard *et al.*^1997^), and in one instance from a soldier severely injured by a land mine (Hernandez, Ramisse and Ducoureau 1998) and from a pulmonary infection (Ghelardi *et al.*^2007^). However, none of these were biopesticidal isolates. The *konkukian* strain isolated from the wounded soldier can be reliably placed in the *anthracis* clade, as can the RM1 strain isolated from lung tissue (ST386)—a group known for its ability to infect vertebrates (Fig. 1) (Raymond and Bonsall 2013). In one report, a farmer developed a corneal ulcer after being splashed in the face with Dipel, a *Bt kurstaki* product, and *Bt* was recovered from that ulcer (Samples and Buettner 1983). However, in that incident the farmer applied a corticosteroid lotion to his eye before the ulcer developed. Corticosteroids can suppress the immune system and delay wound healing, so in this case the *Bt* spores may have simply persisted in the eye without being the main cause of infection (Siegel 2001). The EFSA opinion also cites Helgason *et al.* (2000a), claiming it shows that *Bt* was found associated with periodontal infections. In fact, that study identified only one isolate of *Bt*, which was recovered from a dairy farm and not a human infection (Helgason *et al.*2000a). A second study cited described how two *Bt* strains were recovered from the blood of immunecompromised patients, but the genotyping scheme used could not confirm they were biopesticidal strains (Kuroki *et al.*^2009^).

The EFSA opinion takes a very uncritical interpretation of key data in the *Bacillus* literature. In the 39 food-poisoning outbreaks studied by McIntyre *et al.* (2008), *Bt* occurred in food consumed in four of these outbreaks (10%) based on detection of *cry* genes and crystals. Although *Bt* could be recovered from food, it was never found in clinical stool specimens, unlike *Bc* (McIntyre, *et al.*^2008^). Given the high prevalence of *Bt* on plants and on sprayed crops, and the expectation that *Bt* would have to replicate in the gut in order to cause infection (Ceuppens *et al.*2012b), it cannot be concluded that *Bt* was the causative agent from these data. We would therefore disagree with EFSA Opinion's interpretation of this article as describing ‘*B. thuringiensis* related food poisonings’ (p 22, para 3.2.2). In the earlier study by Jackson *et al.* (1995), stools from 18 individuals during a food-poisoning outbreak were examined, and in 4 of these people the samples had crystals and a phage type characteristic of *Bt*, but also the presence of a more plausible etiological agent, Norwalk virus. Neither study provided data nor was it claimed that the *Bt* strains identified caused the outbreaks or were from *Bt* biopesticides. In summary, all cases in which *Bt* was recovered from infection are associated with immune suppression, either as the result of burning, massive trauma or medical treatment and there is no convincing evidence that any of the strains studied were the cause of diarrhoea.

Despite the abundance of studies and data produced over the past 50 years supporting the safety of *Bt*, the EFSA opinion makes the startling claim that the ‘actual contribution of the two species [*B. cereus* and *B. thuringiensis*] to gastro-intestinal and non-gastrointestinal diseases in currently unknown’. The basis for this statement is that clinical laboratories do not routinely screen *Bc* isolates for Cry inclusion bodies, and therefore it is not known whether these infections were caused by *Bt* or *Bc.* This claim ignores the substantial data sets on clinical *Bc* group infections that have emerged since the application of multilocus sequence typing (MLST) (Maiden *et al.*^1998^; Priest *et al.*^2004^). MLST schemes for *Bc* vary, but the original scheme used seven loci, covering 2838 bp of housekeeping genes widely distributed across the genome (Priest *et al.*^2004^). While MLST techniques are being replaced by whole-genome sequencing, the older methods are still a sensitive tool for distinguishing chromosomal genotypes and have yielded substantial databases on thousands of isolates over more than 10 years.

Most importantly for this discussion, the key *Bt* biopesticide strains have recognisable sequence types (STs) that are not shared with any known *Bc* strains (Table 1). Given the levels of biopesticide spores in food and in the environment, if biopesticidal *Bt* strains were causing infections we would expect to see their chromosomal STs in clinical infections. Queries of the *B. cereus* pubMLST website (http://pubmlst.org/bcereus/), which defined the above STs (Jolley and Maiden 2010), or the combined SUPERCAT *B. cereus* database (Tourasse, Okstad and Kolstø. 2010) has not identified a single case, to date, where a clinical infection or case of diarrhoea was associated with one of the *Bt* biopesticide STs. While there is still some ambiguity about the appropriate ST of the *Bt aizawai* strain in the product XenTari, no *aizawai* strain has ever been associated with a vertebrate infection. The SUPERCAT database contains data on 2341 isolates, 490 of which have been recovered from vertebrate infections or which carry the pX01 or pX02 *anthracis* virulence plasmids.

**Table 1.** The STs associated with the major *Bt* serovars used in insect pest management are all recovered from insect and environmental sources. Unique sequence ST numbers are defined here according to unique allele profiles in the MLST scheme developed by Priest *et al.* (2004) and hosted by pub.mlst.org. Origins of isolates matching the allelic profiles of these biopesticidal strains were explored: all were recovered from or environmental material (plants, soil), none were recovered from human clinical studies. Total strains in the pub.mlst database: 2095: 18 from diarrhoea; 42 from faeces; 47 blood; 5 vomit; 9 respiratory tract; 7 wound. These STs were matched to the broader SuperCAT database that compiles information from all the available MLST schemes of the *B. cereus* group as well as whole genome data (http://mlstoslo.uio.no). Information on the origin and characteristics of isolates were determined from the above databases or from references listed for isolates.

Product names	*Bt* serovar	Isolate synonyms	ST	Isolates with identical allele profile in SuperCAT (and pub.mlst) databases
DiPel BMP 123 Thuricide	*kurstaki*	HD-1	8	79 (74)
XenTari, Florbac,	*aizawai*	T07033/HD227	15^a^	8 (7)
Novodor	*morrisoni*	BGSC4AA1 biovar. *tenebrionis*	23	23 (21)
Tekar, VectoBac, Aquabac	*israelensis*	BGSC4Q1,ONR60A, H-14, ATCC 35 646	16^b^	6
Tekar, VectoBac,	*israelensis*	BGSC4Q7 HD1002	16	21 (13)

a Not confirmed: other *aizawai* STs include 53, 54, 833, 834.

b Closest match based on available allelic profile: gmk 7; ilv7; pta 2; pur 6; pyc 8; tpi 13.

Again, we can cast our net more broadly to determine if any STs described as *Bt* have ever been associated with clinical infections. Here we focus on isolates in clade 2, as those in the ‘*anthrax’* clade or clade 1 can already be assumed more dangerous for vertebrates (Fig. 1). In clade 2, there are only a few genotypes that have been recovered from both clinical sources and described by others as *Bt* (*pakistani* ST18 and *darmstadiensis* ST56) as well as a *Bc* genotype corresponding to *Bt* HD-771 described by Tourasse *et al.* (2006). Other genotypes initially reported as being comprised of mixtures of Cry producers and non-producers (Raymond *et al.*2010b) have subsequently proven to be mixtures of different genotypes (B. Raymond unpublished data). Thus, only a handful of isolates have a genotype potentially associated with Cry inclusions in one context and in another context of infecting humans. It is entirely plausible that possession of Cry toxin-bearing plasmids was a transient or recent occurrence in these genotypes and that infections in humans were associated with clones that lack Cry toxin synthesis. Database entries are also subject to error. These genotypes and clones are certainly not well studied, and published reports on these genotypes contain no details about their origins or how strains were typed as *Bt*.

In summary, we have a great deal of data on whether or not *Bt* genotypes are associated with clinical infections. The fact that not one of the numerous clinical infections associated with *Bc sensu lato* has ever been found to be caused by biopesticide genotype confirms the results of decades of safety testing and city-wide epidemiological studies. The fact that only a very small number of clinical isolates subject to MLST testing have ever been shown to be genotypically indistinguishable from clones also described as *Bt* should also give us confidence.

### Phylogeny is a better indicator of infection risk for vertebrates than carriage of enterotoxin genes

Some *Bc* specialists, as well as the EFSA opinion, make the argument that *Bt* could be dangerous to vertebrates because these bacteria carry haemolytic enterotoxin genes that are thought to be responsible for the ability of *Bc* to cause diarrhoea (Granum and Lund 1997; Stenfors Arnesen, Fagerlund and Granum 2008; EFSA 2016). It is very important to note that it is the emetic *Bc* strains that cause the most serious cases of food poisoning due to the production of the distinct cereulide toxin, and these strains are largely restricted to a narrow set of lineages and no *Bt* strain have ever been found to be capable of producing cereulide (Agata, Ohta and Mori 1996; Thorsen *et al.*^2006^; Vassileva *et al.*^2007^) (Fig. 1). The argument that *Bt* strains are dangerous because they carry enterotoxins does not hold up to scrutiny. First, most if not all biopesticide strains such as those based on *Bt kurstaki* HD-1 carry enterotoxin genes and score positively on ELISA assays for these proteins, but this is not associated with the ability to infect vertebrates (Damgaard 1995; Bishop, Johnson and Perani 1999). The evidence linking possession of enterotoxin genes to clinical risk is also circumstantial. Enterotoxin gene profiles vary considerably across the *Bc* group (Cardazzo *et al.*^2008^). Haemolytic toxins tend to be absent from lineages containing *B. mycoides* and *B. weihenstephanensis* (Cardazzo *et al.*^2008^), which is consistent with the view that these are non-pathogenic environmental groups (Raymond *et al.*2010b; Raymond and Bonsall 2013) (Fig. 1). Gastrointestinal simulation experiments failed to demonstrate enterotoxin production during growth conditions mimicking that in the ileum (Ceuppens *et al.*2012a), and none of the four major classes of enterotoxin genes are critical for infection in insects (Klimowicz, Benson and Handelsman 2010). Nevertheless, regulation of enterotoxin genes is complex. The presence of one enterotoxin gene may be required for food-poisoning potential but the possession of even multiple genes is not sufficient to indicate the ability to cause intestinal infections in vertebrates (Cardazzo *et al.*^2008^).

In fact, phylogenetic affiliation within the *Bc* is a much better indication of ecological niche or food-poisoning risk (Guinebretière *et al.*^2010^; Raymond *et al.*2010b; Raymond and Bonsall 2013). While some of earlier literature argues that the *Bc* group is homogeneous (Helgason *et al.*2000b) or that *Bc, Bt* and *B. anthracis* should be considered one single species (Tourasse *et al.*^2006^), this most certainly does not represent a consensus. In fact, application of MLST has led to the opposing consensus view: that there are substantial genetic and biological differences between clades in the *Bc* group (Siegel 2001; Priest *et al.*^2004^; Sorokin *et al.*^2006^; Vassileva *et al.*^2006^; Cardazzo *et al.*^2008^; Didelot *et al.*^2009^; Raymond *et al.*2010b; Raymond and Bonsall 2013), while also recognising that these clades do not neatly correspond to given species names. Analyses of the patterns of horizontal gene transfer suggest that there are at least three major clades and that most recombination occurs within rather than between clades (Didelot *et al.*^2009^). One recent whole-genome analysis suggests breaking up the group into 19 or 20 species might be justified (Liu *et al.*^2015^), a recommendation that is perhaps a step too far. Importantly, the major MLST clades have different patterns of host association or varying ability to cause food poisoning (Fig. 1) (Guinebretière *et al.*^2010^; Raymond *et al.*2010b; Raymond and Bonsall 2013). Since *Bt* strains (producing Cry toxins) are widely distributed in several clades, not all *Bt* strains would be expected to be equally safe, as the discussion above suggests. Strains more closely related to *anthracis* are more commonly associated with vertebrate infections, giving us an expectation of greater risk. These phylogenetic analyses support the data from acute safety tests demonstrating that *B. anthracis* is at least one million times more dangerous to vertebrates than biopesticidal stains of *Bt* (Siegel 2001). Critically, lineages containing the biopestide strains from the serovars *israelensis, morrisoni (strain tenebrionis), kurstaki* and *aizawai* are unlikely to be associated with human infection (Fig. 1). The latest information available in the SuperCat database confirm these earlier analyses: 72% of the 548 isolates in the *anthracis* clade (clade 1 or cluster III) either closely resemble *B. anthracis* itself or have been associated with vertebrate infections, while only 29% of the 866 isolates in clade 2 are associated with vertebrate infections. This variation in host range and ecology between different clades within the *Bc* group was inadequately discussed in the EFSA review.

### The production of Cry toxins is ecologically and biologically significant

The fact that Cry toxins are plasmid encoded (and can therefore move between distantly related lineages) is almost certainly the reason why *Bt* and *Bc* do not form tidy, distinct species*.* Nevertheless, the different names can still be useful because the ecological and biological consequences of producing Cry toxins are profound. Since *Bc* enterotoxins can be degraded by stomach acids and digestive enzymes, it is thought that their presence in verterbrate infections must be the result of new vegetative growth in the lower intestine (Ceuppens *et al.*2012b). A key barrier to infection success in *Bc* is competition with existing gut microbes (Ceuppens *et al.*2012b). The carriage of Cry toxin plasmids substantially weakens the competitive ability of *Bt* relative to that of *Bc in vivo* (Raymond, Davis and Bonsall 2007; Raymond *et al.*^2012^) and in soil (Yara, Kunimi and Iwahana 1997). Poor competitive ability is likely to make *Bt* substantially less fit in the gut of vertebrates, where Cry toxin production is not adaptive. While entomocidal *Bt* strains appear to have specific adaptations that enable them to compete effectively with aerobic intestinal microbes in the invertebrate gut (Raymond *et al.*^2008^, ^2009^), the production of Cry toxin, or the carriage of Cry toxin-bearing plasmids, may explain the reduced ability to cause infections in the vertebrate intestine.

### Conclusion

To summarise, the recent controversial case of food poisoning in Germany presents no convincing evidence that *Bt* was the causative agent, since individuals with food poisoning had also consumed a dose of *Bc* sufficient to cause the observed level of infection. Overall, the MLST databases, the epidemiological studies and safety testing literature present a well-informed and coherent view of the biology and ecology of the *Bc* group. The arguments in the EFSA report, that we do not understand the risks of consuming *Bt* spores, are therefore unfounded and overly cautious. An analysis of studies cited in EFSA's opinion used to question *Bt* safety (Rosenquist *et al.*^2005^, Frederiksen *et al.*^2006^) show not only do humans routinely eat high levels of this species, but that most of the strains (>80%) consumed are naturally occurring, not from biopesticides. Yet even at rates not considered acceptable under Danish guidelines, there is no evidence that consumption has ever resulted in food poisoning. Furthermore, strains of entomocidal *Bt* are not capable of infecting vertebrates at extremely high doses in controlled laboratory tests and there are no robust data to suggest that humans might be an exception. Phylogenetic analyses of ecological differentiation across the *Bc* group suggest that there are very few strains of *Bt* with elevated risks for vertebrates (Guinebretière *et al.*^2010^; Raymond *et al.*2010b; Raymond and Bonsall 2013). This would include the subsp. *konkukian*, which was originally isolated from a soldier severely injured by a land mine (Hernandez, Ramisse and Ducoureau 1998). That isolate did indeed pose a greater risk to mice than biopesticidal strains of *Bt* (Hernandez *et al.*^2000^). Crucially, the *Bt konkukian* can be firmly placed in the *anthracis* clade and is distantly related to all the biopesticidal strains (Han *et al.*^2006^; Raymond *et al.*2010b; Raymond and Bonsall 2013); it is also not demonstrably pathogenic to insects. Based on the ecological differentiation across the *Bc* group, we would not recommend licensing any *Bt* products that show a similar biological affinity to *B. anthracis.*

Regulators do not have a particularly easy job. For plant protection products, be they chemical or biological, it is never possible to eliminate risk entirely. Making the argument that we do not know enough to assure governments of a reasonable level of safety is therefore tempting. Recommendations for greater levels of precaution can always be justified. However, a highly cautious approach has consequences in terms of the ever-narrower range of products available to growers or the increasing costs associated with pest management. Without doubt, we do need to control pests, but ever greater levels of restriction are likely to make the horticultural and agricultural economy of the European Union increasingly uncompetitive. Moreover, tighter restrictions on the use of *Bt* products will mean that growers will return to the use of registered broad-spectrum synthetic chemical insecticides, which without question are more harmful to the environment. Regulators must therefore carefully weigh the balance of evidence before urging greater restrictions. For *Bt*, there is simply no case for increasing restrictions on this valuable, highly safe biological insecticide.
